# Increased Efficacy of Oral Fixed-Dose Combination of Amphotericin B and AHCC^®^ Natural Adjuvant against Aspergillosis

**DOI:** 10.3390/pharmaceutics11090456

**Published:** 2019-09-03

**Authors:** Alba Pérez-Cantero, Dolores R. Serrano, Patricia Navarro-Rodríguez, Andreas G. Schätzlein, Ijeoma F. Uchegbu, Juan J. Torrado, Javier Capilla

**Affiliations:** 1 Unitat de Microbiologia, Facultat de Medicina i Ciències de la Salut, Universitat Rovira i Virgili and Institut d’Investigació Sanitària Pere Virgili (IISPV), 43201 Reus, Tarragona, Spain; 2 Departament of Pharmaceutics and Food Technology and Instituto Universitario de Farmacia Industrial (IUFI), School of Pharmacy, University Complutense, Avenida Complutense, 28040 Madrid, Spain; 3 UCL School of Pharmacy, 29–39, Brunswick Square, London WC1N 1AX, UK; 4 Nanomerics Ltd., St. Albans AL1 1SR, UK

**Keywords:** aspergillosis, amphotericin B, oral delivery, chitosan, shiitake, *Lentinula edodes*, AHCC^®^, Molecular Envelope Technology

## Abstract

Invasive pulmonary aspergillosis represents one of the most serious fungal infections among immunocompromised patients. In this study, we aimed to analyze the in vivo efficacy of prophylactic oral amphotericin B (AMB) encapsulated in modified chitosan-nanoparticles (Nanomerics’ Molecular Envelope Technology (MET)) supplemented with a standardized extract of cultured *Lentinula edodes* mycelia (AHCC^®^) in a murine model of pulmonary aspergillosis. We determined fungal burden and survival of mice and additionally, we carried out a cytokine analysis in an attempt to understand the immunomodulation of the extract. Our results evidenced equivalent efficacy between orally administered AMB-MET and the intravenous liposomal AMB marketed formulation. Addition of the AHCC^®^ supplement significantly improved efficacy in terms of burden reduction and survival increase of both oral and intravenous AMB therapies compared to the untreated control group. Moreover, a protective effect of the extract was observed in terms of weight loss. Regarding the cytokine profiles, the Th1 immune response was stimulated in treated animals when compared to the control group. This response was marked by an enhancement in the MCP-1, GM-CSF, VEGF, RANTES and IL-17 levels and a decrease in the IL-6, a biomarker related to the severity of the infection.

## 1. Introduction

Invasive aspergillosis (IA) is a life-threatening condition that mostly affects immunocompromised patients, including those following stem cell or solid organ transplantation, patients with hematological malignancies, granulomatous disease, and neutropenia, or those under chemotherapy or corticosteroid treatment [1,2]. Within the genus, *Aspergillus fumigatus* is the main causal agent of the condition, with an associated mortality rate that ranges from 50 to 100% depending on the delay in diagnosis and treatment, as well as the underlying state of the patient or the affected organs [3,4].

The current IA treatment guidelines [5] establish voriconazole (VRC) as the treatment of choice, although the increase in the number of azole-resistant isolates observed in recent years has had a direct effect on patient outcomes, leading to therapeutic failure. Other recommended therapies include intravenous liposomal amphotericin B (LAMB), echinocandins or isavuconazole [5,6,7,8], yet other options are being studied in order to achieve better therapeutic outcomes and provide expanded alternatives of treatment. This has resulted in antifungal combinations [9,10], the development of new antifungal agents or improved formulations of the preexisting ones [11].

Amphotericin B (AMB) represented the first-line treatment for IA for many years due to its higher efficacy in treating aspergillosis. However, it is well-known that it can display great nephrotoxicity and cause a broad variety of side effects [12]. These complications are responsible for treatment interruption, which can exacerbate mortality rates or extend patient hospitalization [13,14,15]. Different AMB formulations are currently available for their parenteral use, including LAMB, AMB lipid complex (ABLC) or AMB colloidal dispersion (ABCD), all of them with reduced toxic effects when compared to the conventional micellar deoxycolate AMB formulation (dAMB). Even though these lipidic formulations have improved the efficacy-safety profile of the drug, they still require parenteral administration and, in addition, there are no oral formulations commercially available nowadays, which hampers amphotericin’s use in developing countries [16].

In this context, novel AMB formulations are currently under research in order to achieve safer formulations that could be orally administered and, therefore, improve the patients’ quality of life. Some of these in-development formulations include nanoparticle-based carrier systems [17,18,19]. In this sense, we demonstrated in a previous work the efficacy of oral AMB encapsulated in *N*-palmitoyl-*N*-monomethyl-*N*,*N*-dimethyl-*N*,*N*,*N*-trimethyl-6-*O*-glycol chitosan nanoparticles (AMB-MET) in a murine model of invasive aspergillosis, in which we achieved translocation to specific organs of the pathology, sparing the site of toxicity with a comparable efficacy to LAMB [15].

Despite the advances in the development of new drug delivery systems, the resolution of IA remains challenging, in part due to the immunocompromised state of the patients. Therefore, boosting the immune status of the patients seems a key point in order to satisfactorily treat IA. The use of natural extracts as potential adjuvants that could stimulate and enhance the immune system is emerging as a promising approach for the treatment of different illnesses. For instance, *Lentinula edodes*, the shiitake mushroom, has been traditionally used to treat quotidian aches and aging fatigue [20]. Other properties associated with this mushroom are related to heart health or lung diseases, however, there is an important lack of experimental data [20]. Nevertheless, shiitake extract’s benefits have been demonstrated in mice, as they have been used as adjuvants in hepatitis vaccines [21]. Additionally, chemotherapy-subjected patients have displayed longer survival when shiitake extract has been combined with conventional chemotherapy [22].

One of the most studied extracts of the shiitake mushroom is AHCC^®^ [23]. AHCC^®^ is a standardized cultured extract of shiitake mycelia, manufactured by a proprietary mycelia cell culture process, as its components differ according to the culture conditions [24]. In particular, AHCC^®^ is mainly composed of carbohydrates such as acetylated α-1,4 glucan, but also contains proteins, minerals, fats, and fiber [25], and it has been suggested to play an important role in the orchestration of the immune response to viral, parasitic, and bacterial infections, as well as the maintenance of immune system homeostasis [26]. Consequently, the aim of the present study was to evaluate the in vivo efficacy of oral AMB-MET supplemented with AHCC^®^ in a murine model of invasive pulmonary aspergillosis as a potential prophylactic therapy.

## 2. Materials and Methods

### 2.1. Materials

The AMB was purchased from Azelis (Barcelona, Spain). The AHCC^®^ was kindly provided by Amino Up Co., Ltd. (Sapporo, Japan). The AmBisome^®^ was supplied by Gilead Sciences S.L. (Madrid, Spain). The sodium deoxycholate was supplied by Fluka Chemie AG (Buchs, Switzerland). All other reagents and chemicals were obtained from Sigma Aldrich Chemical Co. (Poole, UK or Madrid, Spain), Panreac S.A. (Barcelona, Spain), Fisher Scientific (Loughborough, UK) or VWR (Lutterworth, UK). The AMB-MET nanoparticles were prepared and characterized as previously reported [15].

### 2.2. Animals

A total of 75 OF-1 four-week-old male mice weighing 35 g (Charles River, Criffa S.A. Barcelona, Spain) were divided into groups of 15 animals/group, with 10 used for the survival studies and 5 for fungal load determination. Animals were housed under standard conditions with water and food ad libitum. All procedures were supervised and approved by the Universitat Rovira i Virgili Animal Welfare and Ethics Committee (URV reference 0247, 15 March 2017).

### 2.3. Inoculum

One *A. fumigatus* clinical strain (FMR 7738) isolated from blood was cultured at 37 °C for 5 days on potato dextrose agar (PDA) plates prior to its use. The inoculum was prepared by flooding the culture plate with 5 mL of saline with 0.05% Tween^®^ 20, with the application of gentle agitation to release the conidia. Afterward, the conidial suspension was serially diluted and adjusted to the desired concentration by a hemocytometer count.

### 2.4. Murine Model of Pulmonary Aspergillosis

A murine model of pulmonary aspergillosis was used as previously described [27]. Immunosuppression of mice was established 4 days before infection and every 3 days by subcutaneous injection of cortisone acetate at 125 mg/kg [28]. On the day of infection, mice were anesthetized with inhaled sevoflurane and inoculated by nasal instillation with 25 µL of a suspension containing 5 × 10^4^ conidia. Animals were checked twice daily until the end of the experiment (11 days post-infection).

### 2.5. Treatment

Animals received prophylactic treatment starting 5 days before infection, followed by therapeutic treatment. Prophylaxis consisted of AMB-MET administered orally by gavage (p.o) at 2.5 mg/kg twice a day (BID) or intravenous (i.v.) LAMB at 2.5 mg/kg BID. Both drugs were administered alone or in combination with AHCC^®^ administered p.o. twice a day at 270 mg/kg. On the day of infection, no compound was administered. One day after infection and for 11 days, the animals received oral AMB-MET at 5 mg/kg p.o. BID or LAMB at 5 mg/kg i.v. BID, both alone or in combination with oral AHCC^®^ administered BID at 270 mg/kg. The control animals received only the vehicle (PBS).

### 2.6. Fungal Burden Studies

Six days after the infection, the animals from the tissue burden group were anesthetized by inhaled sevoflurane. The blood was obtained by cardiac puncture and the serum was collected after blood centrifugation and frozen until used for further analysis. In addition, 11 days post-infection, the surviving mice from the mortality study groups were also subjected to cardiac puncture in the same manner. In all cases, immediately after blood collection, the animals were euthanized by cervical dislocation, and brain, kidneys, and lungs were aseptically removed, weighed, and mechanically homogenized in 1 mL of sterile saline. Homogenates were serially 10-fold diluted, placed onto PDA plates and incubated at 35 °C for Colony Forming Unit (CFU) determination.

### 2.7. Sera Determinations

The AMB levels were determined from all obtained sera by bioassay as previously described [29] using *Candida parapsilosis* ATCC 22019 as the control strain, but also by a validated HPLC method [30]. Briefly, the AMB was isocratically eluted using a Thermo^®^ Hypersil BDS C18 reverse-phase column (200 × 4.6 mm, 5 μm) and a mobile phase consisted of acetonitrile, acetic acid, and water (52:4.3:43.7, *v*/*v*/*v*) with a flow rate of 1 mL min^−1^. The absorbance was monitored at 406 nm and the injection volume was set at 40 µL. The cytokine quantification was performed from the animal sera samples consisting of 100 µL from the control (6 and 11 days post-infection) and the treatment groups (11 days post-infection) using a Quantibody^®^ array (RayBiotech, Norcross, GA, USA, distributed by BioNova cientifica, Madrid, Spain). This multiplexed sandwich ELISA-based quantitative array platform was able to analyze: GM-CSF (granulocyte-macrophage colony-stimulating factor), IFN-γ (interferon), IL-1a, IL-1b, IL-2, IL-3, IL-4, IL-5, IL-6, IL-9, IL-10, IL-12, IL-13, IL-17, KC (chemokines), MCP (monocyte chemoattractant protein)-1, M-CSF (macrophage colony-stimulating factor), TNF-α (tumor necrosis factor), RANTES (regulated on activation, normal T cell expressed and secreted), and VEGF (vascular endothelial growth factor) using a pair of cytokine specific antibodies for detection. A capture antibody was first bound to the glass surface and after incubation with the sample, the target cytokine was trapped on the solid surface. A second biotin-labeled detection antibody was then added, which could recognize a different epitope of the target cytokine. The cytokine-antibody-biotin complex was visualized through the addition of the streptavidin-conjugated Cy3 equivalent dye using the GenePix^®^ 4000B Scanner (Unidad de Genomica, Campus Moncloa, UCM, Madrid, Spain).

### 2.8. Statistical Analysis

The fungal burden results were analyzed by the Mann–Whitney U test and the survival curves were compared among them by the log-rank test using GraphPad Prism 6.0 for Windows (GraphPad Software Inc; La Jolla, CA, USA).

The statistical differences in the sera AMB levels were evaluated via a one-way ANOVA test using Minitab 15 (Minitab Ltd., Coventry, UK), Tukey’s test was used for paired-group comparisons.

The cytokine statistical analysis was performed using both a univariate and multivariate technique as previously reported [31]. The normality test (Shapiro–Wilk) was performed. The Kruskal–Wallis pairwise comparison test was carried out when the *P*-value was < 0.05 while a one-way ANOVA (Tukey’s post hoc test) was used for those cytokines that followed a normal distribution (*P* > 0.05). The multivariate data analysis (MVA) was performed using the Unscrambler^®^ X software (CAMO Software, Oslo, Norway). The treatment effect on the cytokine profile was analyzed by principal component analysis (PCA) to study systematic variability and the relationships between variables and scores. The correlation loadings of the principal components (PCs) represented the variance for each variable for a given PC, giving information about the variability source inside the dataset. *P*-values ≤ 0.05 were considered as statistically significant.

## 3. Results

### 3.1. Survival and Body Weight Progression

**Figure 1:**
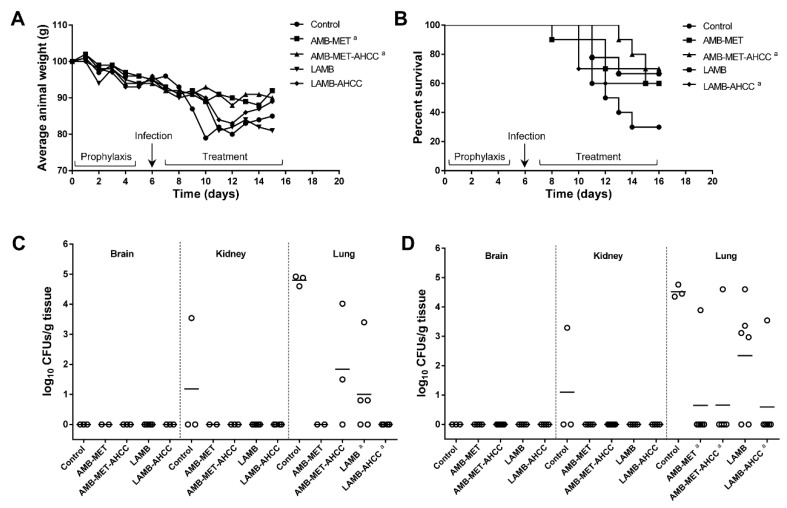
Effect of AMB-MET p.o. and LAMB i.v. with and without AHCC^®^ supplementation in immunosuppressed mice infected intranasally with *Aspergillus fumigatus*. (**A**) Progression of body weight. (**B**) Percent of surviving mice. Panels (**C**,**D**) show scattergram and median (horizontal lines) of CFUs recovered from targeted organs at 6 and 11 days after infection, respectively. ^a^ Indicates statistically significant differences (*P* < 0.05) in comparison to the control group.

The pulmonary infection resulted in elevated mortality in animals from the control group. The untreated animals began to die six days after challenge and at the end of the experiment (day 11 post-infection), only 30% had survived to the infection (Figure 1A). Oral AMB-MET or i.v. LAMB alone did not increase, in a significant manner, the survival rates in comparison to the control group (*P* = 0.171 and 0.130, respectively). However, a statistically significant beneficial effect was observed in terms of survival when oral AMB-MET and i.v. LAMB were administered in combination with oral AHCC^®^ (*P* = 0.033 and 0.029, respectively, in comparison to the control group). No significant differences were observed between the LAMB-AHCC^®^ and AMB-MET-AHCC^®^ treatments (*P* = 0.530).

Regarding the animals’ weight, which was checked daily starting five days before the infection and until the end of the experiment, the results showed a considerable decrease (15%) in the untreated mice (Figure 1B). The LAMB-treated mice were the group with the most significant weight loss (19.2%), while the supplementation of the treatment with AHCC^®^ only resulted in an 11% reduction. The AMB-MET and AMB-MET-AHCC^®^ were the treatments associated with a minor weight reduction, with an 8.5% and 9.5% average weight loss (*P* ≤ 0.0068), respectively, indicating good gastrointestinal tolerance after oral administration.

### 3.2. Fungal Burden

The tissue burden study performed six days post-infection showed poor dissemination of *Aspergillus* to the brain and kidneys in control animals, while a high amount of fungus was recovered from the lung tissue (4.80 ± 0.17 log_10_ CFUs/g) (Figure 1C). The AMB-MET and LAMB monotherapies reduced the CFUs from the lungs (0 and 1.00 ± 0.46 log_10_ CFUs/g, respectively), in a significant manner with respect to the control animals (both *P* = 0.018). Similarly, the addition of AHCC^®^ reduced the fungal load from the lungs in those animals receiving LAMB (*P* = 0.0286), although this significant effect was not observed in those treated with AMB-MET (*P* = 0.06) in comparison to the control group. No significant differences in the tissue burden reduction were found between the monotherapies (*P* = 0.4286) or between the two AHCC^®^-combined therapies (*P* = 0.1429).

By day 11 post-infection, no fungal loads were detected in the brain of the control or treated groups of animals, and only one kidney from the control group was positive for fungal presence. The lungs remained highly affected in the untreated animals (4.52 ± 0.21 log_10_ CFUs/g) (Figure 1D). At this time, LAMB therapy was unable to significantly reduce the tissue burden when compared to the controls (*P* = 0.083). However, the combination of LAMB with AHCC^®^ resulted in a great lung burden reduction (*P* = 0.0119) with 83% of animals displaying infection clearance. Similarly, high clearance was obtained in the animals receiving AMB-MET with or without AHCC^®^ (86% and 83% infection clearance, respectively) and this reduction was statistically significant in comparison with the untreated group (*P* = 0.0119 and 0.0250, respectively). No statistically significant differences were observed among the various treatment groups (*P* ≥ 0.197).

### 3.3. AMB Concentration in Plasma

**Figure 2:**
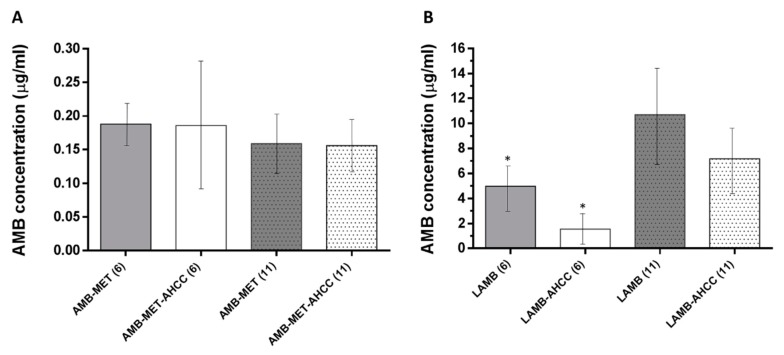
AMB concentration in plasma after day 6 (6) and day 11 (11) post-infection. (**A**) Animals treated with AMB-MET with and without AHCC^®^. (**B**) Animals treated with LAMB with and without AHCC^®^. * *P*-value< 0.05 Day 6 vs. day 11 post-infection.

The AMB levels in plasma were significantly higher for those animals treated with LAMB compared to AMB-MET (*P* < 0.05) (Figure 2). The administration of AHCC^®^ as an immunoadjuvant did not alter in a significant manner the AMB concentration in the plasma in any of the formulations. Additionally, no differences were observed in the AMB plasma levels in those animals treated with AMB-MET at day six and day 11 post-infection. However, the AMB levels were significantly greater at day 11 compared to day six for LAMB.

### 3.4. Cytokine Analysis

**Figure 3:**
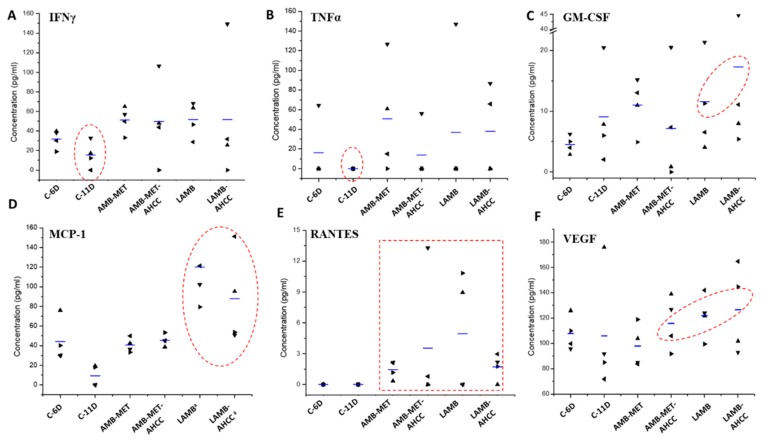
Scattergram of cytokine levels at day 6 and 11 for control group and at day 11 post-infection for all treatment groups. Horizontal lines indicate mean values of (**A**) IFNγ, (**B**)TNFα, (**C**) GM-CSF, (**D**) MCP-1, (**E**) RANTES, (**F**) VEGF. Key: C-6D, control group at day six post-infection, C-11D, control group at day 11 post-infection, AMB-MET, and AMB-MET-AHCC, treatment with AMB encapsulated within GCPQ nanoparticles alone or in combination with AHCC^®^ respectively, LAMB and LAMB-AHCC, treatment with i.v. liposomal AMB alone or in combination with AHCC^®^ respectively. The most relevant changes in the cytokine patterns have been highlighted within a red circle. ^a^ *P* < 0.05.

**Figure 4:**
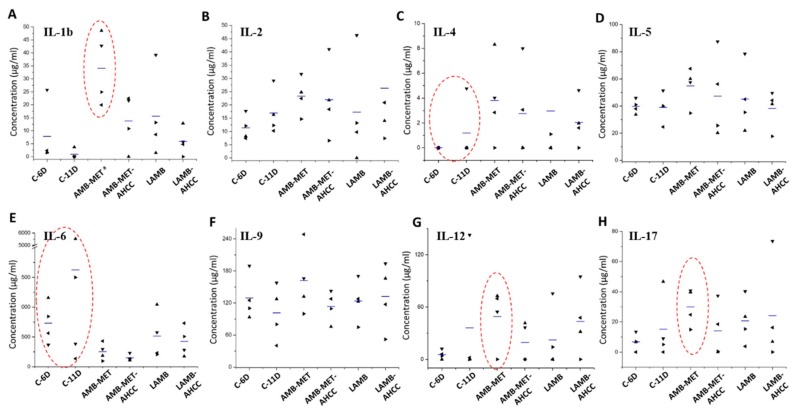
Scattergram of cytokine at day 6 and 11 for control group and at day 11 post-infection for all treatment groups. Horizontal lines indicate mean values of (**A**) IL-1b, (**B**) IL-2, (**C**) IL-4, (**D**) IL-5, (**E**) IL-6, (**F**) IL-9, (**G**) IL-12, (**H**) IL-17. Key: C-6D, control group at day six post-infection, C-11D, control group at day 11 post-infection, AMB-MET, and AMB-MET-AHCC, treatment with AMB encapsulated within GCPQ nanoparticles alone or in combination with AHCC^®^ respectively, LAMB and LAMB-AHCC, treatment with iv liposomal AMB alone or in combination with AHCC^®^ respectively. The most relevant changes in the cytokine patterns have been highlighted within a red circle. ^a^ *P* < 0.05.

In general, all treatments showed the tendency to increase the production of the cytokines IFN-γ, TNF-α, GM-CSF, RANTES, VEFG, IL-4, IL-17, IL-2, and especially MCP-1 (*P* = 0.002) and IL-1b (*P* = 0.043), while the rest of the cytokines were diminished or maintained in comparison to the control group (Figure 3 and Figure 4). In the case of the animals treated with LAMB, greater levels of GM-CSF, MCP-1 and VEGF were found compared to the control group, while administration of AMB-MET led to higher secretion of IL-1b, IL-12, and IL-17. The combination of LAMB-AHCC^®^ increased the levels of IL-2 and IL-12, while in those receiving AMB-MET the administration of AHCC^®^ increased RANTES and VEFG. AHCC^®^ administered in combination with LAMB or AMB-MET diminished the release of IL-1b, IL-4, IL-5, IL-6, and IL-9 in comparison to the monotherapies. It is worth mentioning the slight reduction of IL-6 in the treated animals and especially in those receiving AHCC^®^, since IL-6 is a biomarker of the infection severity (*P* = 0.059). The secretion of IL-12, IL-17, TNF-α, and GM-CSF was higher in those animals receiving AMB-MET-AHCC^®^ than in the animals treated only with AMB-MET. Contrarily, these cytokines were detected at equal or slightly higher concentrations in animals treated with LAMB-AHCC^®^ when compared to the LAMB group.

**Figure 5:**
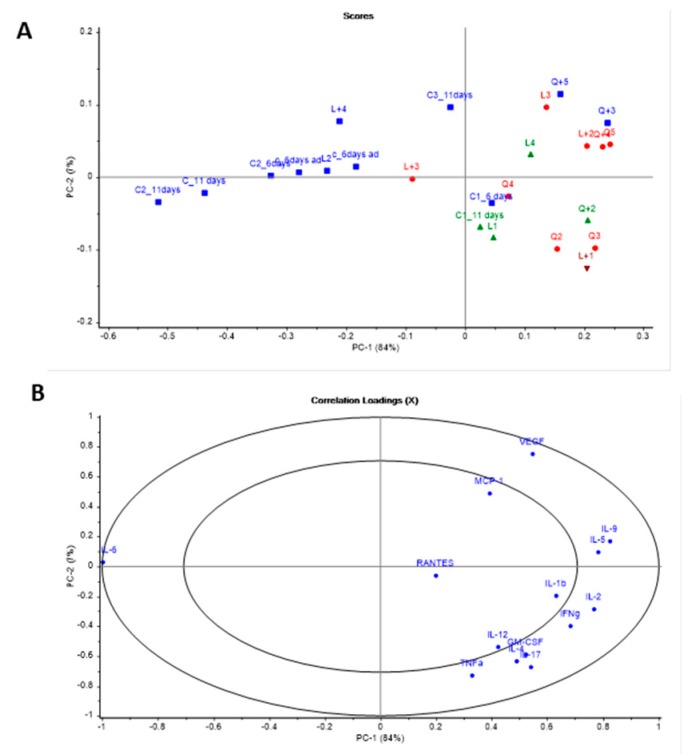
Principal component analysis of the cytokine profile at day six and 11 for control group and at day 11 post-infection for all treatment groups. (**A**) Score plot. (**B**) Correlation loading plot. Key: C represents animals from the untreated control group; L- animals treated with LAMB and Q- animals treated with AMB-MET.

The PCA (Figure 5) showed that most of the variance found in the cytokine analysis was due to two principal components (84% for PC1 and 7% for PC2). When the samples were grouped based on the PCA, the majority of controls were grouped together (blue color) while most of the treated animals were grouped within each other (red color). However, some differences were observed, as the other two groups (green and brown) were also differentiated and shared common features (Figure 5A). Regarding the correlation loading plots, the IL-6 response was located completely opposed to the markers of a positive prognosis, such as RANTES, IL-2 or IFN-γ (Figure 5B). Additionally, an antagonistic effect was observed between the responses of VEGF and MCP-1 (higher with the LAMB treatment) versus Il-12 and IL-17 (enhanced in the AMB-MET group).

## 4. Discussion

The discovery of new options for the treatment of IA is essential in order to overcome the therapeutic failure associated with this condition. Therapeutic failure is mostly due to the increase of azole resistant *Aspergillus* isolates and the toxicity events associated with the current therapies [32,33]. With the difficulties and limitations linked to the development of new antifungal drugs and the finding of new safe-to-human targets, establishing new therapeutic and prophylactic approaches with the already available drugs is crucial.

In this study, we aimed to test the in vivo efficacy of a recently engineered chitosan encapsulated AMB nanoformulation, AMB-MET [15], supplemented with the natural extract AHCC^®^ from the mushroom *Lentinula edodes* as prophylactic treatment in order to clarify and characterize its role as an adjuvant. The supplementation with AHCC^®^ has already shown benefits in the treatment of melanoma in animal models [34]. Other researchers have also found similar effects when administering AHCC^®^ alone or in combination with drugs destined to treat other conditions, such as gastric cancer, but also when combined with their chemotherapy treatment in murine models [22]. In the field of infectious diseases, different studies have demonstrated a beneficial effect of AHCC^®^, specifically against canine leishmaniosis [35], influenza [36], *Chlamydia trachomatis* infections [37], and West Nile encephalitis in mouse models [38]. In addition, the positive effects against bacterial infections have been described [39].

Regarding our study, the in vivo efficacy of the treatments we assayed (AMB-MET and LAMB) against aspergillosis has already been established by others [15,40,41]. Nevertheless, the results we found in terms of survival in all of the treated animals revealed a significantly higher efficacy of the assayed drugs when supplemented with the natural extract AHCC^®^, in comparison to the untreated group, which evidences a clear beneficial effect of the natural extract. As we also compared the fungal burden of the treated and untreated animals at days six and 11 post-infection, we found a burden reduction in the lung tissue of all the assayed treatments at both experimentation points. Surprisingly, at day six, the AMB-MET+AHCC^®^, LAMB, and LAMB+AHCC^®^ treatments reduced the burden, in a significant manner. At day 11, this was achieved by AMB-MET, AMB-MET+AHCC^®^, and LAMB+AHCC^®^, suggesting that AMB-MET may have a wider activity in the long-term. This could be due to the preferential distribution in the lung tissue after oral administration of AMB-MET [15] and the higher toxicity of i.v. LAMB when administered for prolonged periods of time, which might have also led to a significant weight loss in the treated animals of this group. Along with the AHCC^®^ adjuvant role on efficacy, we found that this extract acted as a protective agent of weight loss and appetite diminution, which is very well exemplified in the weight loss differences between the LAMB and LAMB+AHCC^®^ therapies, accompanied by an average weight loss of 19.2% and 10%, respectively. Several nutritional effects have already been linked to this kind of glucans, such as the reduction of stress and cholesterol levels, hypoglycemic effects or improvement of ulcerative colitis cases [42].

**Figure 6:**
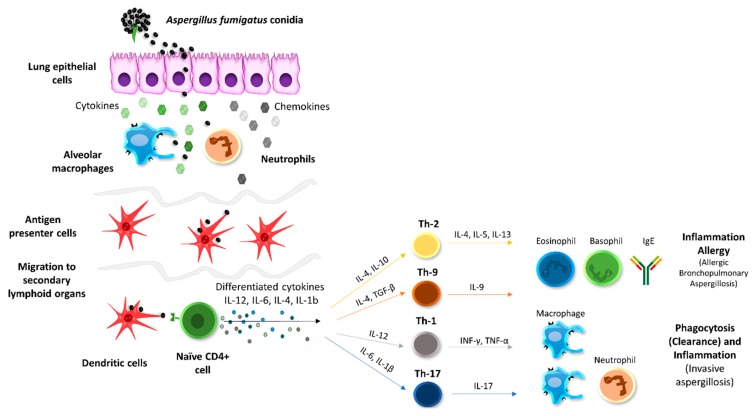
Schematic representation of immunological response against *A. fumigatus* antigens and production of differentiated cytokines and effector T_H_-cells (adapted and modified from [45]).

Besides, the protective role of AHCC^®^ in infectious diseases seems to be related to its immunomodulatory properties by priming TLR-4 and TLR-2 [26] and the associated TLR-4/MyD88 and NF-κB/MAPK signal transduction pathways [43]. In this sense, an increase in MCP-1 secretion by intestinal epithelial cells and an increase in both IL-1b and TNFα secretion by monocytes have been reported as the key effect after administration of AHCC^®^ [43]. In addition, AHCC^®^ has previously been shown to potentially promote macrophage and natural killer cell proliferation [43,44]. On this basis, we performed a cytokine analysis to better characterize the immunomodulatory effects of AHCC^®^ in our treatment groups against aspergillosis. A schematic representation of the immunological response associated to *A. fumigatus* infections is illustrated in Figure 6.

In the case of fungal infections, the outcome partly depends on the Th1 protective cellular response, which is principally driven by the proinflammatory cytokines TNF, IFN, IL-6, IL-12, and IL-1. In contrast, the Th2 response is not protective but regulates the excessive inflammatory conduction of Th1. This response is characterized by the increase in the cytokines IL-4, IL-5, IL-13, and the promotion of non-opsonizing antibodies. Other cytokines also related to fungal infections are IL-8, IL-10, and IL-15, as well as the already mentioned IL-17 [46,47,48]. Interestingly, in our work, most of the studied cytokines were found to be higher in those animals receiving any treatment in comparison to the control group, with the exception of IL-6, a biomarker related to the severity of the infection, which was accordingly reduced in the treated animals. Our results also showed a slight increase in the Th2 response in the treated animals that, combined with the increase of the Th17 response promoted by IL-17, can display a positive effect due to its anti-inflammatory and tissue protection properties [46].

Furthermore, increased levels of MCP-1, GM-CSF, and VEGF, which have been associated with favorable outcomes in infectious diseases by promoting monocyte attraction, macrophage maturation and killing [49,50,51], were also found in our treated groups. In addition, both the AMB-MET and LAMB treated animals showed an increase in IFN-γ levels compared to the control group, which is correlated with a better prognosis since IFN-γ activates the immune system response through the stimulation of macrophages and neutrophils [52]. Similar results were obtained for TNF-α, even though only a few animals from the treated groups displayed a response for this biomarker. The increase in TNF-α is also associated with a more positive response towards microorganisms, since it stimulates the protective Th1 response [53]. Likewise, we observed increased levels of monocyte chemotactic protein (MCP-1), especially in the LAMB group, which has a favorable role in pulmonary infections due to its association with the attraction of more monocytes to the site of infection and the promotion and maturation to macrophages, and hence, MCP-1 facilitates microorganism eradication [49].

Another biomarker that can be used in systemic fungal infections is RANTES. RANTES levels, which drop drastically in sick individuals with systemic fungal infections [50], were reestablished in our treated animals, while its levels were correspondingly undetectable in the control group.

In the case of the GM-CSF, its levels were slightly increased in the AMB-MET and LAMB groups, which was a positive response, considering that higher values of GM-CSF are associated with macrophage maturation, stimulation and, therefore, with improved innate immune response [49]. Finally, with regard to VEGF, slightly greater levels were found in the LAMB group compared to the other treatments. Promisingly, previous reports on pulmonary aspergillosis models have associated higher values of VEGF with greater animal survival [51]. We are aware of the limitations of the present study due to the low number of surviving animals in the sampling days (i.e., day six and 11 post-infection), which together with the high dispersion of data prevent us from robustly linking the measured cytokines levels with the observed benefit of using AHCC^©^.

Taken together, our data suggest that oral administration of AMB-MET matches the efficacy of i.v. liposomal AMB against *Aspergillus*, being of great advantage to be used for immunocompromised patients discharged from hospital. Moreover, the combination of AMB with AHCC^®^, with positive effects at six and 11 days post-infection, seems to play an extremely important role as an adjuvant by boosting the immune system response and protecting against weight loss and appetite diminution.

## 5. Conclusions

Our in vivo study results evidenced high efficacy against aspergillosis of AMB-MET and LAMB therapies when combined with the AHCC^®^ supplement since, in contrast to the AMB-based monotherapies, AMB-MET/LAMB with AHCC^®^ supplementation displayed statistically significant improvements with respect to the control group in terms of survival and burden reduction. Although the AHCC^®^ immune system boost is very promising, further studies on its immunomodulation and its potential use as an adjuvant with other antifungals against invasive fungal infections need to be carried out.
